# Pre-Treatment with Laminarin Protects Hippocampal CA1 Pyramidal Neurons and Attenuates Reactive Gliosis Following Transient Forebrain Ischemia in Gerbils

**DOI:** 10.3390/md18010052

**Published:** 2020-01-12

**Authors:** Tae-Kyeong Lee, Ji Hyeon Ahn, Cheol Woo Park, Bora Kim, Young Eun Park, Jae-Chul Lee, Joon Ha Park, Go Eun Yang, Myoung Cheol Shin, Jun Hwi Cho, Il-Jun Kang, Moo-Ho Won

**Affiliations:** 1 Department of Neurobiology, School of Medicine, Kangwon National University, Chuncheon 24341, Gangwon, Korea; xorud312@naver.com (T.-K.L.); nbrkim17@gmail.com (B.K.); taeparo@naver.com (Y.E.P.); anajclee@kangwon.ac.kr (J.-C.L.); 2 Department of Biomedical Science, Research Institute of Bioscience and Biotechnology, Hallym University, Chuncheon 24252, Gangwon, Korea; jh-ahn@hallym.ac.kr; 3 Leefarm Co., Ltd., Hongcheon 25117, Gangwon, Korea; flfhflfh@naver.com; 4 Department of Anatomy, College of Korean Medicine, Dongguk University, Gyeongju 38066, Gyeongbuk, Korea; jh-park@dongguk.ac.kr; 5 Department of Radiology, Kangwon National University Hospital, Chuncheon 24289, Gangwon, Korea; yangke@kangwon.ac.kr; 6 Department of Emergency Medicine, School of Medicine, Kangwon National University, Chuncheon 24341, Gangwon, Korea; dr10126@naver.com (M.C.S.); cjhemd@kangwon.ac.kr (J.H.C.); 7 Department of Food Science and Nutrition, Hallym University, Chuncheon 24252, Gangwon, Korea

**Keywords:** gliosis, ischemia-reperfusion, polysaccharide, pyramidal neurons, prevention, rodents

## Abstract

Transient brain ischemia triggers selective neuronal death/loss, especially in vulnerable regions of the brain including the hippocampus. Laminarin, a polysaccharide originating from brown seaweed, has various pharmaceutical properties including an antioxidant function. To the best of our knowledge, few studies have been conducted on the protective effects of laminarin against ischemic injury induced by ischemic insults. In this study, we histopathologically investigated the neuroprotective effects of laminarin in the Cornu Ammonis 1 (CA1) field of the hippocampus, which is very vulnerable to ischemia-reperfusion injury, following transient forebrain ischemia (TFI) for five minutes in gerbils. The neuroprotective effect was examined by cresyl violet staining, Fluoro-Jade B histofluorescence staining and immunohistochemistry for neuronal-specific nuclear protein. Additionally, to study gliosis (glial changes), we performed immunohistochemistry for glial fibrillary acidic protein to examine astrocytes, and ionized calcium-binding adaptor molecule 1 to examine microglia. Furthermore, we examined alterations in pro-inflammatory M1 microglia by using double immunofluorescence. Pretreatment with 10 mg/kg laminarin failed to protect neurons in the hippocampal CA1 field and did not attenuate reactive gliosis in the field following TFI. In contrast, pretreatment with 50 or 100 mg/kg laminarin protected neurons, attenuated reactive gliosis and reduced pro-inflammatory M1 microglia in the CA1 field following TFI. Based on these results, we firmly propose that 50 mg/kg laminarin can be strategically applied to develop a preventative against injuries following cerebral ischemic insults.

## 1. Introduction

A transient brain ischemic insult, caused by temporary insufficiency of blood supply to the brain, inflicts ischemia-reperfusion injury to the brain [1,2]. Among these ischemic insults, transient forebrain ischemia (TFI) occurs due to the occlusion of both common carotid arteries and triggers selective neuronal death/loss in particularly vulnerable subregions of the forebrain including the striatum, neocortex and hippocampus [3,4]. For example, the pyramidal neurons in the stratum pyramidale of the Cornu Ammonis 1 (CA1) field of the hippocampus die four to five days after a brief TFI (a five-minute TFI) in gerbils [3,5,6]. Because the death of CA1 pyramidal neurons occurs four to five days after TFI, this phenomenon is referred to as “delayed neuronal death” [5]. In response to the delayed neuronal death following TFI, glial cells (astrocytes and microglia) proliferate or undergo hypertrophy; this is known as “reactive gliosis” [3,7].

Laminarin (LA) is a β-glucan type of polysaccharide, and it originates from marine Phaeophyta (brown algae) [8,9]. LA has been widely researched because of its bioactive attributes. For example, LA enhances anti-cancer immunity via maturation of dendritic cells [8]. Additionally, LA improves immune responses via promoting T- and B-cell and macrophage populations in leukemic mice [10]. Several recent studies regarding LA have shown that LA reduces H_2_O_2_-induced oxidative stress via regulation of the Nrf2 pathway in human lung fibroblast (MRC-5) cells [11]. Also, LA improves the quality of aged porcine oocytes by alleviating oxidative stress [12].

However, to the best of our knowledge, the protective effects of LA against brain ischemic insults have not been adequately studied. The purpose of this study was to investigate the neuroprotective effect of LA following TFI in gerbils, which are regarded as good animal models of TFI [3,7,13,14]. Also, we examined the influence of LA on reactive gliosis (astrogliosis and microgliosis) following TFI.

## 2. Results

### 2.1. Neuroprotection by LA

#### 2.1.1. Cresyl Violet (CV) Stained Cells

**Figure 1:**
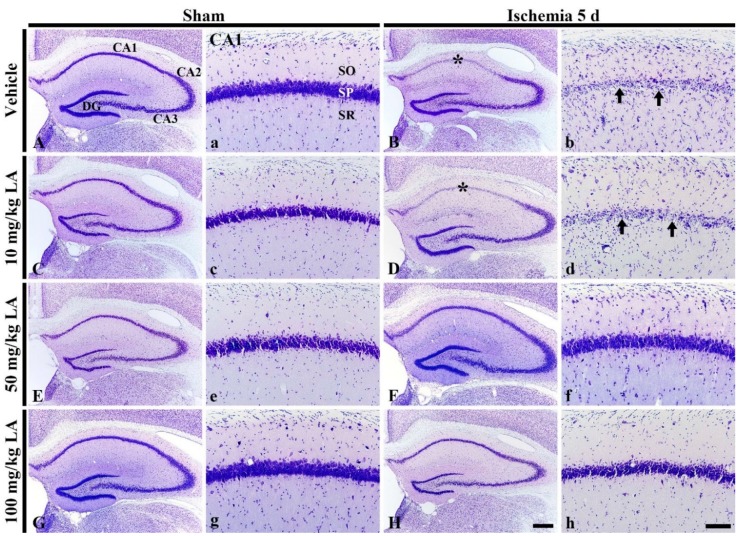
Cresyl Violet (CV) staining in the hippocampus (**A**–**H**) and its Cornu Ammonis 1 (CA1) field (**a**–**h**) of the vehicle/sham (**A**,**a**), 10, 50 and 100 mg/kg laminarin (LA)/sham (**C**,**c**,**E**,**e**,**G**,**g**), vehicle/ischemia (**B**,**b**) and 10, 50 and 100 mg/kg LA/ischemia (**D**,**d**,**F**,**f**,**H**,**h**) groups at 5 days after sham or transient forebrain ischemia (TFI) operation. In the vehicle/ischemia group, CV dyeability is remarkably reduced in the stratum pyramidale (SP, arrows) of the CA1 field (asterisks). In the 10 mg/kg LA/ischemia group, the distribution pattern of CV stained cells is similar to that in the vehicle/ischemia group. However, in the 50 mg/kg and 100 mg/kg LA/ischemia groups, CV stainability is conserved. DG, dentate gyrus; SO, stratum oriens; SR stratum radiatum. Scale bars = 400 μm (**A**–**H**) and 100 μm (**a**–**h**).

In the vehicle/sham group, all hippocampal subfields were well stained by CV and pyramidal cells, which are located in the stratum pyramidale, were clearly detected by CV staining (Figure 1A,a). The distribution pattern of CV-stained cells in this group was not different from that in the normal group (data not shown). In all of the LA-sham groups, the distribution of CV-stained cells in the hippocampus was similar to that of the vehicle/sham group (Figure 1C,c,E,e,G,g).

In the vehicle/ischemia group, the distribution pattern of CV-stained cells was altered compared to the vehicle/sham group (Figure 1A,B). Namely, the dyeability of CV was markedly reduced in the stratum pyramidale of the CA1 field in the hippocampus proper (CA1-3 fields) (Figure 1a,b). Additionally, in the stratum oriens and radiatum, CV-stained cells were greatly increased compared to those in the vehicle/sham group (Figure 1a,b).

In the LA/ischemia groups, the distribution pattern of CV-stained cells in the CA1 field of the 10 mg/kg LA/ischemia group was very similar to that in the vehicle/ischemia group (Figure 1B,b,D,d). This finding indicates that treatment with 10 mg/kg LA cannot protect pyramidal cells in the CA1 field, which are called CA1 pyramidal cells or neurons. In contrast, in the 50 mg/kg and 100 mg/kg LA/ischemia groups, CA1 pyramidal cells were well stained with CV like those in the vehicle/sham group (Figure 1F,f,H,h). This finding indicates that treatment with 50 mg/kg or 100 mg/kg LA can protect neurons from TFI.

#### 2.1.2. Neuronal-Specific Nuclear Protein (NeuN) Immunoreactive Neurons

**Figure 2:**
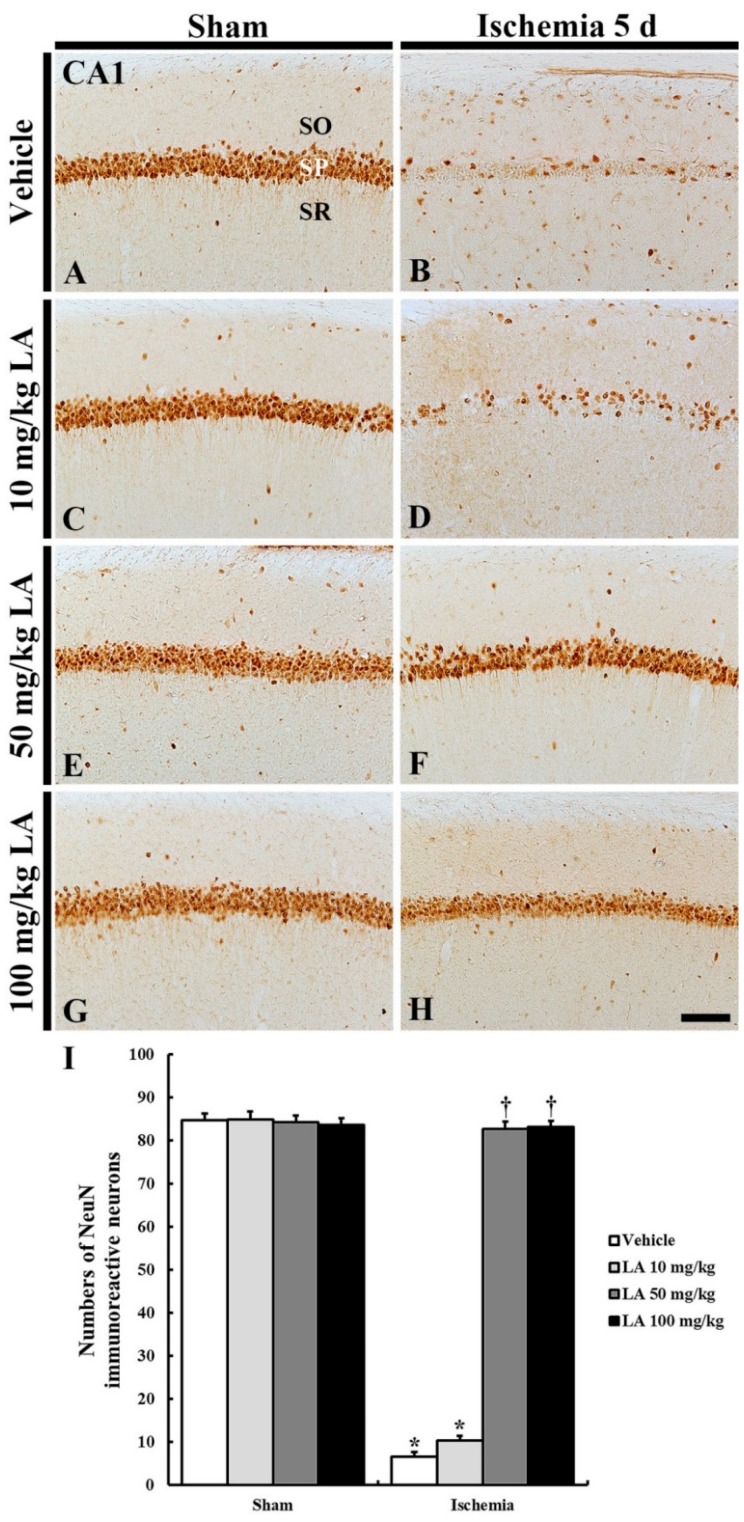
NeuN immunohistochemistry in the CA1 field of the vehicle/sham (**A**), 10, 50 and 100 mg/kg LA/sham (**C**,**E**,**G**), vehicle/ischemia (**B**) and 10, 50 and 100 mg/kg LA/ischemia (**D**,**F**,**H**) groups at 5 days after sham or TFI operation. Numerous NeuN immunoreactive CA1 pyramidal neurons can be observed in the vehicle/sham group. In the vehicle/ischemia and 10 mg/kg LA/ischemia groups, significant decreases in NeuN immunoreactive CA1 pyramidal neurons were detected. In the 50 mg/kg and 100 mg/kg LA/ischemia groups, CA1 pyramidal neurons show strong NeuN immunoreactivity. Scale bar = 100 μm. (**I**) Mean number of NeuN immunoreactive pyramidal cells in the CA1 field at 5 days after TFI (*n* = 7 in each group, * *p* < 0.05 versus vehicle/sham group, † *p* < 0.05 versus vehicle/ischemia group). The bars indicate the means ± SEM.

In all the sham groups, NeuN immunoreactive CA1 pyramidal neurons were easily detected, and the number of NeuN immunoreactive CA1 pyramidal neurons was not significantly different in all of the sham groups (Figure 2A,C,E,G,I).

In the vehicle/ischemia group, NeuN immunoreactive CA1 pyramidal neurons were rarely observed in the stratum pyramidale, and the number of NeuN immunoreactive neurons was significantly reduced compared to that of the vehicle/sham group (Figure 2B,I).

In the 10 mg/kg LA/ischemia group, NeuN immunoreactive CA1 pyramidal neurons were rarely found, and the number of NeuN immunoreactive CA1 pyramidal neurons was no different to that in the vehicle/ischemia group (Figure 2D,I). However, NeuN immunoreactive CA1 pyramidal neurons were well observed in the 50 mg/kg and 100 mg/kg LA/ischemia groups, and the number of NeuN immunoreactive CA1 pyramidal neurons was significantly increased compared to that in the vehicle/ischemia group (Figure 2F,H,I).

#### 2.1.3. Fluoro-Jade B (F-J B) Positive Cells

**Figure 3:**
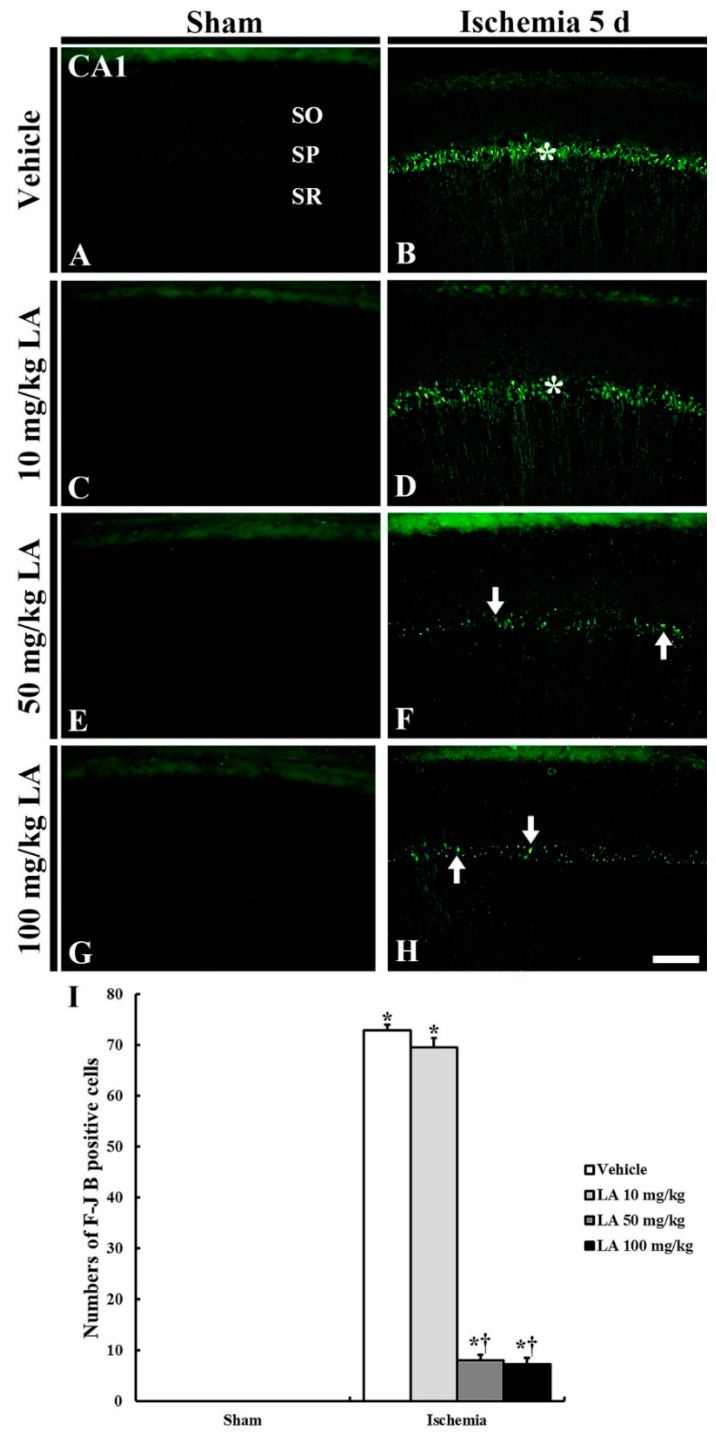
F-J B histofluorescence staining in the CA1 field of the vehicle/sham (**A**), 10, 50 and 100 mg/kg LA/sham (**C**,**E**,**G**), vehicle-ischemia (**B**) and 10, 50 and 100 mg/kg LA/ischemia (**D**,**F**,**H**) groups at 5 days after sham or TFI operation. In all the sham groups, no F-J B positive cells are found in the CA1 field; numerous F-J B positive cells are shown in the SP (asterisks) in the vehicle/ and 10 mg/kg LA/ischemia groups. However, in the 50 mg/kg and 100 mg/kg LA/ischemia groups, F-J B positive cells (arrows) are significantly decreased. Scale bar = 100 μm. (**I**) Mean number of F-J B positive pyramidal cells in the CA1 field at 5 days after TFI (*n* = 7 in each group, * *p* < 0.05 versus vehicle/sham group, † *p* < 0.05 versus vehicle/ischemia group). The bars indicate the means ± SEM.

No F-J B positive cells, which are dead cells, were detected in the CA1 field of all the sham groups (Figure 3A,C,E,G,I).

In the vehicle/ischemia group, numerous F-J B positive cells were shown in the stratum pyramidale of the CA1 field (Figure 3B,I). This finding means that CA1 pyramidal neurons died following the 5-min TFI.

In the 10 mg/kg LA/ischemia group, the pattern of F-J B fluorescence was similar to that of the vehicle/ischemia group, and the number of F-J B positive cells was not different from the vehicle/ischemia group (Figure 3D,I). However, in the 50 mg/kg and 100 mg/kg LA/ischemia groups, a few F-J B positive CA1 pyramidal cells were found, and the number of F-J B positive cells was about 11% and 10%, respectively, (*p* < 0.05) of that in the vehicle/ischemia group (Figure 3F,H,I).

### 2.2. Attenuation of Gliosis by LA

#### 2.2.1. Glial Fibrillary Acidic Protein (GFAP) Immunoreactive Astrocytes

**Figure 4:**
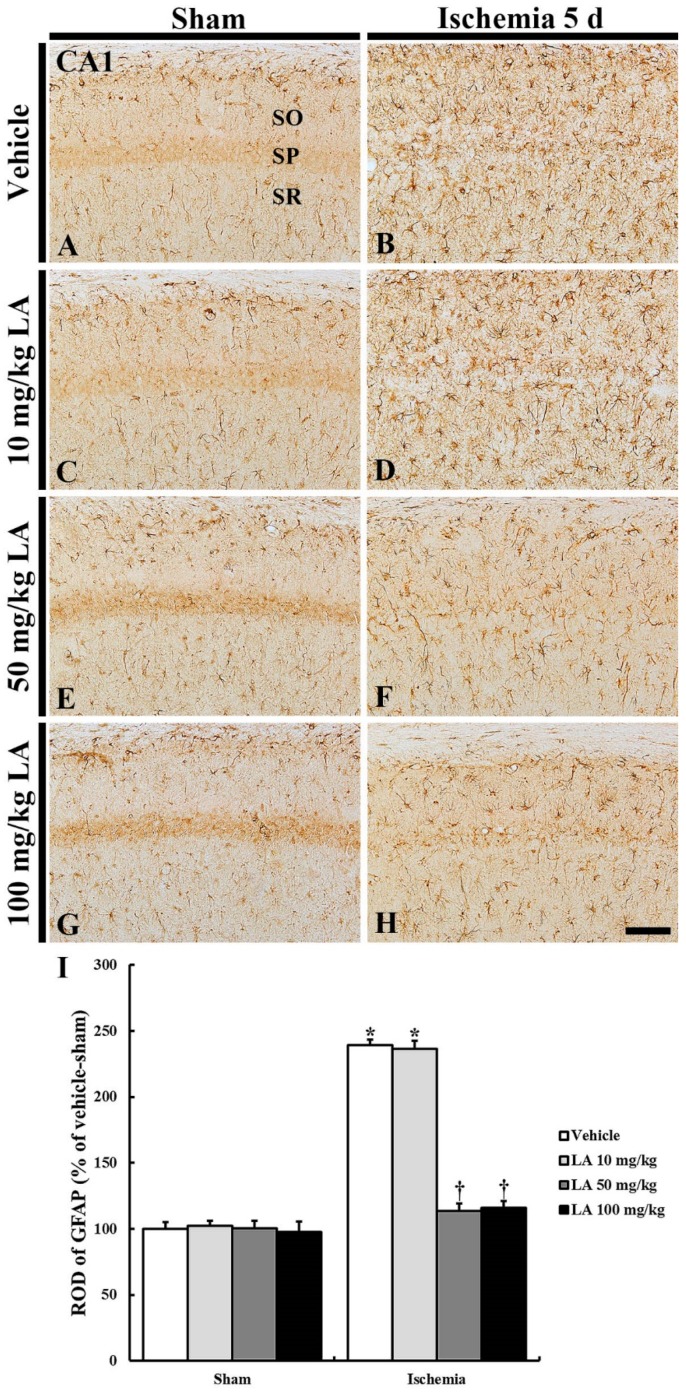
Glial fibrillary acidic protein (GFAP) immunohistochemistry in the CA1 field of the vehicle/sham (**A**), 10, 50 and 100 mg/kg LA/sham (**C**,**E**,**G**), vehicle/ischemia (**B**) and 10, 50 and 100 mg/kg LA/ischemia (**D**,**F**,**H**) groups at 5 days after sham or TFI operation. In all the sham groups, typical GFAP immunoreactive astrocytes are generally distributed in the stratum oriens (SO) and radiatum (SR). In the vehicle/ischemia group, GFAP immunoreactive astrocytes are hypertrophied. In the 10 mg/kg LA/ischemia group, GFAP immunoreactive astrocytes are similar to those in the vehicle/ischemia group. In the 50 mg/kg and 100 mg/kg LA/ischemia groups, hypertrophy of GFAP immunoreactive astrocytes is apparently attenuated. Scale bar = 100 μm. (**I**) ROD (percentage) of GFAP immunoreactive structures in the CA1 field at 5 days after TFI (*n* = 7 in each group, * *p* < 0.05 versus vehicle/sham group, † *p* < 0.05 versus vehicle/ischemia group). The bars indicate the means ± SEM.

In all the sham groups, GFAP immunoreactive astrocytes in the CA1 field were shown to be in a resting state and were predominantly distributed throughout the stratum oriens and radiatum in the CA1 field (Figure 4A,C,E,G).The GFAP immunoreactivity of the astrocytes was shown to be similar in all the groups (Figure 4I).

In the vehicle/ischemia group, GFAP immunoreactive astrocytes were hypertrophied, and their processes were thickened (Figure 4B). In this group, GFAP immunoreactivity was significantly increased (239% of the vehicle/sham group) (Figure 4I).

In the 10 mg/kg LA/ischemia group, the distribution and relative optical density (ROD) of GFAP immunoreactive astrocytes were similar to those in the vehicle/ischemia group, that is, the ROD was 236% of the vehicle/sham group (Figure 4D,I). However, in the 50 mg/kg and 100 mg/kg LA/ischemia groups, hypertrophy of immunoreactive astrocytes was apparently attenuated compared to the vehicle/ischemia group (Figure 4B,D,F,H).ROD was significantly reduced to 113% and 116%, respectively, of the vehicle/sham group (Figure 4I).

#### 2.2.2. Ionized Calcium-Binding Adapter Molecule 1 (Iba-1) Immunoreactive Microglia

**Figure 5:**
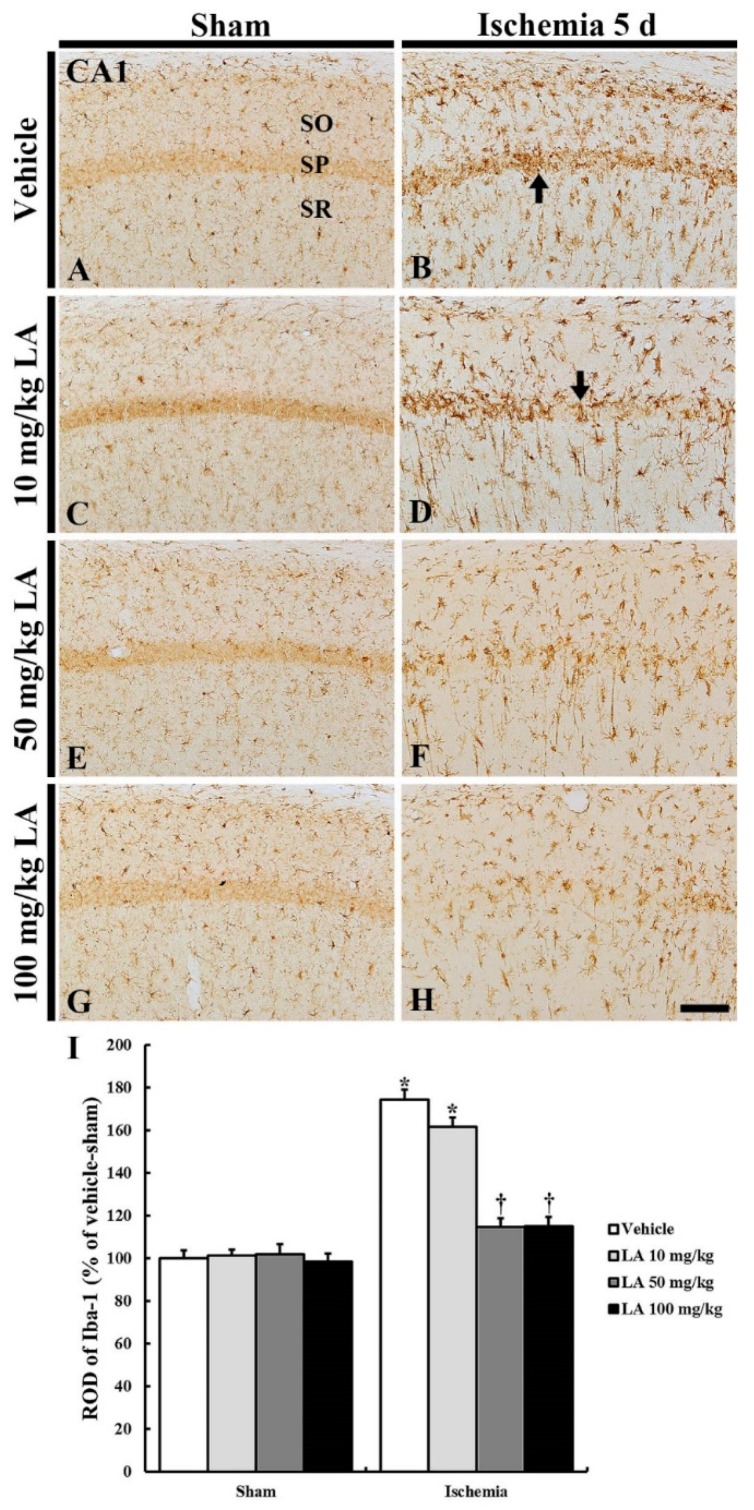
Ionized calcium-binding adapter molecule 1 (Iba-1) immunohistochemistry in the CA1 field of the vehicle/sham (**A**), 10, 50 and 100 mg/kg LA/sham (**C**,**E**,**G**), vehicle/ischemia (**B**) and 10, 50 and 100 mg/kg LA/ischemia (**D**,**F**,**H**) groups at 5 days after sham or TFI operation. Iba-1 immunoreactive microglia are in a resting state in all the sham groups. In the vehicle/ischemia and 10 mg/kg LA/ischemia groups, Iba-1 immunoreactive microglia are hypertrophied, showing that many activated microglia gather in the SP (arrows). In the 50 mg/kg and 100 mg/kg LA/ischemia groups, activation of Iba-1 immunoreactive microglia is markedly attenuated, showing that they are evenly distributed in the CA1 field. Scale bar = 100 μm. (**I**) ROD (percentage) of Iba-1 immunoreactive structures in the CA1 field at 5 days after TFI (*n* = 7 in each group, * *p* < 0.05 versus vehicle/sham group, † *p* < 0.05 versus vehicle/ischemia group). The bars indicate the means ± SEM.

In all the sham groups, Iba-1 immunoreactive microglia in a resting state were uniformly and generally distributed in the stratum oriens and radiatum in the CA1 field (Figure 5A,C,E,G). There was no significant difference in their distribution among all the groups (Figure 5I).

In the vehicle/ischemia group, Iba-1 immunoreactive microglia were hypertrophied (activated), and many of them gathered in the stratum pyramidale where CA1 pyramidal neurons were degenerating or dead (Figure 5B). In addition, Iba-1 immunoreactivity (ROD) in this group was significantly stronger (174% of the vehicle/sham group) than that in the vehicle/sham group (Figure 5I).

In the 10 mg/kg LA/ischemia group, the distribution pattern of Iba-1 immunoreactive microglia was fundamentally similar to that in the vehicle/ischemia group; the ROD of the Iba-1 immunoreactive microglia was lower than that in the vehicle/ischemia group (Figure 5D,I). However, in the 50 mg/kg and 100 mg/kg LA/ischemia group, hypertrophy (activation) of Iba-1 immunoreactive microglia was markedly reduced compared to that in the vehicle/ischemia group (Figure 5B,D,F,H), and the ROD in each group was 114% and 115%, respectively, of the vehicle/sham group (Figure 5I).

#### 2.2.3. Interleukin 2 (IL-2) Immunoreactive Microglia

**Figure 6:**
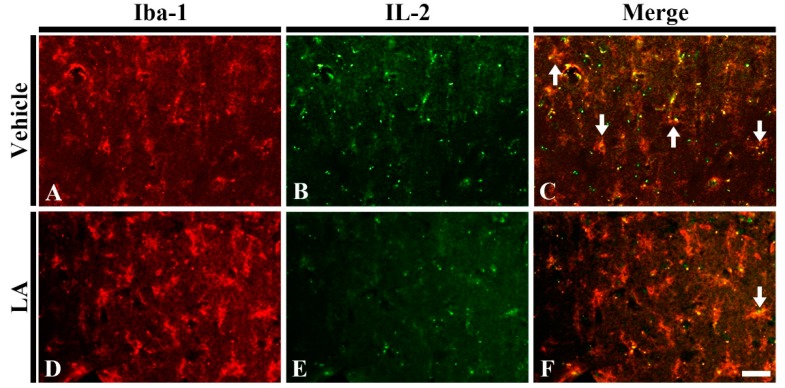
Double immunofluorescence staining for Iba-1 (red), interleukin 2 (IL-2) (green) and merged images in the hippocampal CA1 field of the vehicle/ischemia (**A**–**C**) and 50 mg/kg LA/ischemia (**D**–**F**) groups at 5 days after TFI. Many IL-2 immunoreactive microglia (arrows) are shown in the vehicle/ischemia group. However, in the 50 mg/kg LA/ischemia group, a few IL-2 immunoreactive microglia are detected. Scale bar = 40 μm (*n* = 7 in each group).

To investigate whether LA reduced pro-inflammatory M1 microglia following TFI, we carried out double immunofluorescence staining for IL-2 as pro-inflammatory cytokine and Iba-1 in the vehicle/ischemia and 50 mg/kg LA/ischemia groups. In the vehicle/ischemia group, many of microglia were colocalized with IL-2 (Figure 6A–C). On the other hand, in the 50 mg/kg LA/ischemia, the number of microglia with IL-2 was dramatically reduced in the ischemic CA1 field (Figure 6D–F). This finding indicates that pretreatment with laminarin decreases pro-inflammatory M1 microglia following TFI.

## 3. Discussion

The death or loss of pyramidal neurons in the hippocampal CA1 field is easily induced by ligation of both common carotid arteries (bCCA) for five minutes in gerbils, because they lack the posterior communicating arteries consisting of the circle of Willis in the basal part of the brain [3,4]. The death of CA1 pyramidal neurons occurs four-five days after TFI induced by ligation of bCCA, thus, this phenomenon is referred to as “delayed neuronal death (DND)” [5]. In this study, we found that DND of CA1 pyramidal neurons occurred five days after TFI, similar to the findings of Kirino (1982) and other previous studies [1,5,15].

In this study, we applied LA, a polysaccharide originating from brown algae, in doses of 10, 50 and 100 mg/kg before TFI to investigate its neuroprotective effect. Pretreatment with 10 mg/kg of laminarin did not protect CA1 pyramidal neurons from damage by TFI, but pretreatment of 50 or 100 mg/kg of laminarin protected the neurons from damage. Based on this finding, we determined that a dose of at least 50 mg/kg of LA is required to achieve a neuroprotective effect, however, the 100 mg/kg dose of LA was considered an overdose.

To the best of our knowledge, diverse kinds of seaweeds have been investigated because of their neuroprotective effects. For example, extract from *Dictyopteris divaricate*, belonging to the Dictyotaceae family, displays protective effect via regulating expressions of apoptosis-related proteins in an oxygen and glucose deprivation/reperfusion cell culture model using human neuroblastoma cells [16]. Additionally, extract and fractions from *Agarum clathratum*, a family of Agaraceae, show neuroprotective effects by reducing reactive astrogliosis and microgliosis following TFI in gerbils [17]. In particular, Kang et al. (2012) reported that fucoidan, a sulfated polysaccharide derived from brown seaweeds, displayed neuroprotective effects in a rat model of lipopolysaccharide-accelerated ischemic injury following middle cerebral artery occlusion through inhibition of cytokine expression and neutrophil infiltration [18]. Additionally, Kim et al. (2019) recently reported that fucoidan showed strong neuroprotective properties following TFI in gerbils via amelioration of reactive gliosis and enhancement of antioxidant efficacies [19].

A number of previous studies have demonstrated that reactive gliosis due to various pathological conditions in the central nervous system increases permeability of the blood-brain barrier [20,21,22]. This can accelerate ischemic neuronal damage [23] and trigger inflammatory responses [24,25,26], which can exacerbate neuronal death [25]. Reactive gliosis commonly occurs following TFI, and in this process, glial cells (astrocytes and microglia) are hypertrophied with a thickened cellular structure [6,7,15]. Alleviation of reactive gliosis is connected with neuroprotection following cerebral ischemia [1,6,7]. In this study, we pretreated gerbils with 50 or 100 mg/kg of laminarin and found that reactive gliosis was significantly attenuated in the CA1 field following TFI.

Glial cells including astrocytes and microglia have various physiological functions in the central nervous system (CNS). Astrocytes form a blood-brain barrier (BBB) between neuronal circuitries and blood vessels, and transport nutrients to neurons by cellular linkage [27]. The BBB controls the water and ion balance via regulating expressions of diverse channels in the astrocyte endfeet [22]. Therefore, the breakdown of the BBB is connected with damaged astrocyte endfeet and can trigger various pathological processes in the CNS, such as brain edema following cerebral ischemia [28,29,30,31]. Thus, the breakdown of BBB increases permeability and causes the infiltration of inflammatory cytokines and cells [30,32,33]. It has been reported that treatment with extract from the fruits of *Lycium barbarum* protects the mouse brain from focal ischemia injury induced by middle cerebral artery occlusion by protecting the BBB from breaking down and reducing reactive astrogliosis [34,35]. *Lycium barbarum* belongs to the Solanaceae family, and the fruits contain polysaccharide [34]. In this regard, the neuroprotection from pretreatment with LA 50 mg/kg and 100 mg/kg in TFI may be due to reducing reactive astrogliosis, which is related to maintaining the integrity of the astrocyte endfeet in the BBB. There are several methodologies available for determining whether drug-like compounds penetrate the BBB via measuring the permeability-surface area product (LogPS) [36]. However, in this study, there is a limitation as we did not determine whether LA crossed the BBB or not. We will study whether LA or its metabolites penetrate the BBB in the future.

Microglia are resident immunocytes of macrophage lineage and present in the CNS of vertebrates [37]. Microglia express numerous pattern-recognition receptors that detect pathogen-associated molecular patterns or tissue damage-associated molecular patterns [38]. Additionally, microglia communicate with neurons to maintain a healthy environment in the CNS against neurotoxicity and neuroinflammation [39]. When the CNS is subject to pathological conditions including TFI, microglia secrete diverse kinds of inflammatory cytokines and clear dead cells and tissue debris [40,41,42]. A recent study showed that polysaccharides from the *Ganoderma lucidum* (a Ganodermataceae family) attenuated microglia-mediated neuroinflammation in BV2 microglia stimulated by lipopolysaccharide (LPS) and in primary mouse microglial cells stimulated by amyloid β_42_ oligomer [43]. Additionally, it has been reported that polysaccharides extracted from *Lycium barbarum* display inhibitory effects on the production of LPS-induced pro-inflammatory mediators such as NF-κB, caspase 3, TNF-α and HSP60 in BV2 cells [44]. Research has demonstrated that pro-inflammatory M1 microglia are predominantly distributed in the hippocampal CA1 field when the hippocampus is under microgliosis induced by TFI [26]. In the present study, pretreatment with 50 mg/kg of LA reduced microglia secreting pro-inflammatory cytokines. Thus, we suggest that, in this study, the attenuation of reactive microgliosis by pretreatment with LA may be related to the attenuation of neuroinflammatory responses that can be triggered by reactive microgliosis.

In conclusion, pretreatment with 50 or 100 mg/kg of LA protected pyramidal neurons in the hippocampal CA1 field after a five-minute TFI in gerbils, indicating that reactive gliosis was significantly attenuated in the ischemic CA1 field. With these findings, we strongly propose that 50 mg/kg of LA can be strategically applied to develop a preventive drug that facilitates protection against cerebral ischemic insults. Additionally, further studies on the mechanisms involved in its protective effect against ischemic insults are required.

## 4. Materials and Methods

### 4.1. Experimental Animals

We used 61 male Mongolian gerbils (6-months old and with a body weight of about 70 g) obtained from the Experimental Animal Center of Kangwon National University (Chuncheon, Gangwon, Republic of Korea). The gerbils were housed under optimal conditions with a suitable room temperature (25 ± 2 °C) and relative humidity (55 ± 5%). A 12 h dark/12 h light cycle was maintained and freely accessible feed and water were provided to the gerbils. The experimental protocol was approved (Approval no., KW-180124-1) by the Institution Animal Care and Use Committee (IACUC) at Kangwon National University. This protocol adhered to the current international law and policy guidelines in the “Guide for the Care and Use of Laboratory Animals” (The National Academies Press, 8th Ed., 2011).

### 4.2. Experimental Groups and Administration of LA

Nine groups were randomly assigned as follows: (1) normal group (*n* = 5); (2) vehicle/sham group (*n* = 7), which was treated with vehicle (sterilized normal saline; 0.85% w/v NaCl) and given the sham TFI operation; (3) vehicle/ischemia group (*n* = 7), which was treated with saline and given the TFI operation; (4), (5) and (6) LA/sham groups (*n* = 21; *n* = 7 in each group), in which each group was treated with LA 10 mg/kg, 50 mg/kg and 100 mg/kg, respectively, and given the sham operation; (7), (8) and (9) LA/ischemia groups (*n* = 21; *n* = 7 in each group), in which each group was treated with LA 10 mg/kg, 50 mg/kg and 100 mg/kg, respectively, and given the TFI operation. Before TFI surgery, vehicle and LA were administered once a day for 7 days via intraperitoneal injection.

### 4.3. Induction of TFI

The surgical procedure for the TFI operation was performed according to our previously published method [45]. In short, the gerbils were anesthetized with a mixture of 2.5% isoflurane gas in 33% oxygen and 67% nitrous oxide [46]. The ventral neck was subsequently shaved and a midline incision was made on the shaved area. Next, bCCA were isolated from connective tissues followed by ligation with non-traumatic aneurysm clips. For reperfusion, the clips were removed at 5 min after bCCA occlusion, and then the incised area was sutured. By using an ophthalmoscope (HEINE K180^®^, Heine Optotechnik, Herrsching, Germany), complete occlusion and reperfusion of the bCCA was verified via observing the stop and recirculation of blood in the central arteries of both retinae. The body temperature of the gerbils was maintained in a normothermic condition (37 ± 0.5 °C) and monitored in real time by using a rectal temperature probe (TR-100) (Fine Science Tools Inc., Foster City, CA, USA). The gerbils in the sham group were subjected to a sham operation, which was conducted using the same surgical procedure but without ligation of both arteries. After the sham or TFI operation, all gerbils were kept in a thermal incubator (Mirae Medical Industry, Seoul, Republic of Korea), which was maintained at 23 °C and 60% of relative humidity.

### 4.4. Tissue Preparation for Histological Examination

The preparation of tissue sections was done according to the method previously described by us [47]. In short, all gerbils were deeply anesthetized by intraperitoneal injection of pentobarbital sodium 70 mg/kg (JW Pharm. Co. Ltd., Seoul, Republic of Korea) [46] at 5 days after the sham or TFI operation. Their brains were rinsed (flow rate, 6 mL/min; total perfused volume, 60 mL) via the ascending aorta with 100 mM phosphate-buffered saline (PBS, pH 7.4) and fixed by perfusion with a solution of 4% paraformaldehyde (in 100 mM phosphate buffer, pH 7.4). Then, the brains were harvested and post-fixed in the same fixative at room temperature for 5 h. Next, for cryoprotection of the brains, the fixed brains were infiltrated with a solution of 30% sucrose (in 100 mM phosphate buffer, pH 7.4) at room temperature for 12 h. Finally, the brains were cut serially into coronal sections of 30 μm thickness in a cryostat (Leica, Nussloch, Germany).

### 4.5. CV Staining

CV staining was carried out in order to examine alterations in cellular morphology and distribution in the hippocampus following TFI as described in our previous paper [3]. Briefly, the brain sections were mounted onto gelatinized microscopy slides. Next, these sections were stained with a solution of 0.1% w/v CV acetate (Sigma, St. Louis, MO, USA) and dehydrated by immersing them in serial ethanol bath. Finally, cover glasses were mounted onto the stained sections with Canada balsam (Kanto, Tokyo, Japan).

### 4.6. F-J B Histofluorescence Staining

To investigate neuronal degeneration or death in the hippocampus due to TFI, we used F-J B, a marker for neuronal degeneration. Histofluorescence staining was conducted as described previously [48]. In brief, the brain sections were reacted with a solution of 0.06% w/v potassium permanganate solution (in distilled water) at room temperature for 20 min. These sections were subsequently immersed in solution of 0.0004% F-J B (Histochem, Jefferson, AR, USA) (in 0.1% glacial acetic acid) at room temperature for 40 min. Then, these sections were dehydrated and mounted with cover glasses by using dibutylphthalate polystyrene xylene (DPX, Sigma, St. Louis, MO, USA) as a mounting medium.

### 4.7. Immunohistochemistry

Immunohistochemistry was performed in order to investigate neuronal loss and reactive gliosis following TFI as previously described in our published paper [1]. In short, the brain sections were reacted with 0.3% H_2_O_2_ (in 10 mM PBS, pH 7.4) at room temperature for 40 min and immersed in 5% normal horse or goat serum (in 10 mM PBS, pH 7.4) at room temperature for 40 min. Then, the treated sections were reacted overnight at 4 °C with each primary antibody: mouse anti-NeuN (1:1000, Chemicon, Temecula, CA, USA) for examining neurons, mouse anti-GFAP (1:1000, Chemicon, Temecula, CA, USA) for examining astrocytes, and rabbit anti-Iba-1 (1:800, Wako, Osaka, Japan) for examining microglia. These immunoreacted sections were subsequently exposed to secondary antibody, which matches each primary antibody as biotinylated horse anti-mouse IgG and (1:250, Vector, Torrance, CA, USA) or biotinylated goat anti-rabbit IgG (1:250, Vector, Torrance, CA, USA). Then, the sections were incubated with avidin-biotin complex (1:300, Vector, Torrance, CA, USA). Finally, these brain sections were visualized by reacting them with a solution of 3, 3′-diaminobenzidine tetrachloride (DAB, Sigma, St. Louis, MO, USA) (in 100 mM PBS, pH 7.4).

### 4.8. Double Immunofluorescence

To examine whether laminarin reduced pro-inflammatory M1 microglia following TFI, double immunofluorescence staining was conducted according to a previously published method [49]. Briefly, the brain sections were incubated with primary antibodies at room temperature for 12 h: rabbit anti-IL-2 (1:50, Santa Cruz, CA, USA) for a pro-inflammatory cytokine and goat-anti Iba-1 (1:150, Abcam, Cambridge, UK) for microglia. Next, the sections were immersed in secondary antibodies at room temperature for 2 h: FITC-conjugated donkey anti-rabbit IgG (1:500, Jackson ImmunoResearch, West Grove, PA, USA) and Cy3-conjugated-donkey anti-goat IgG (1:500, Jackson ImmunoResearch, West Grove, PA, USA). The immunoreactive structures were observed under a confocal microscope (LSM510 META NLO, Carl Zeiss, Göttingen, Germany).

### 4.9. Data Analyses

Data obtained in this study were analyzed according to the methods described in [7]. In brief, NeuN immunoreactive neurons were observed with a light microscope (BX53) (Olympus, Hamburg, Germany), and F-J B positive cells were investigated with an epifluorescent microscope (Carl Zeiss, Göttingen, Germany) equipped with 450–490 nm wavelength of blue excitation light and a barrier filter. Each microscope was equipped with a digital camera (DP72) (Olympus, Hamburg, Germany) connected to a PC monitor. Seven sections per gerbil were selected for cell counting. We captured digital images of NeuN immunoreactive neurons and F-J B positive cells in a 250 × 250 μm square at the center of the hippocampal CA1 field. We conducted cell counting by using a software (Optimas 6.5) (CyberMetrics, Scottsdale, AZ, USA).

To quantitatively analyze GFAP and Iba-1 immunoreactivity, seven sections per animal were chosen. Digital images of GFAP and Iba-1 immunoreactive structures were taken from the target region of the hippocampus by using the same light microscope. The captured images were then calibrated into an array of 512 × 512 pixels, and each immunoreactive image was measured by a 0–255 gray scale system that is, the background density was subtracted, and the ratio of ROD was calibrated by using Adobe Photoshop (ver. 8.0). Finally, ROD was analyzed by using the NIH image (1.59 software). A ratio of ROD was calibrated as a %, with the vehicle/sham group designated as 100%.

### 4.10. Statistical Analysis

Data were statistically presented as the mean ± standard error of mean (SEM). We used SPSS 18.0 software (SPSS, IL, USA), and the data were measured by two-way analysis of variation (ANOVA) with a post hoc Bonferroni’s multiple comparison test in order to express differences among all the experimental groups. Statistical significance was designated as less than 0.05 of *p* value.

## References

[1] Ahn J.H., Shin B.N., Park J.H., Lee T.K., Park Y.E., Lee J.C., Yang G.E., Shin M.C., Cho J.H., Lee K.C. (2019). Pre- and Post-Treatment with Novel Antiepileptic Drug Oxcarbazepine Exerts Neuroprotective Effect in the Hippocampus in a Gerbil Model of Transient Global Cerebral Ischemia. Brain Sci..

[2] Kapoor M., Sharma S., Sandhir R., Nehru B. (2019). Temporal changes in physiological and molecular markers in various brain regions following transient global ischemia in rats. Mol. Biol. Rep..

[3] Lee T.K., Kim H., Song M., Lee J.C., Park J.H., Ahn J.H., Yang G.E., Ohk T.G., Shin M.C., Cho J.H. (2019). Time-course pattern of neuronal loss and gliosis in gerbil hippocampi following mild, severe, or lethal transient global cerebral ischemia. Neural Regen. Res..

[4] Kirino T., Sano K. (1984). Selective vulnerability in the gerbil hippocampus following transient ischemia. Acta Neuropathol..

[5] Kirino T. (1982). Delayed neuronal death in the gerbil hippocampus following ischemia. Brain Res..

[6] Park C.W., Lee T.K., Cho J.H., Kim I.H., Lee J.C., Shin B.N., Ahn J.H., Kim S.K., Shin M.C., Ohk T.G. (2017). Rufinamide pretreatment attenuates ischemia-reperfusion injury in the gerbil hippocampus. Neurol. Res..

[7] Park J.H., Lee T.K., Ahn J.H., Shin B.N., Cho J.H., Kim I.H., Lee J.C., Kim J.D., Lee Y.J., Kang I.J. (2017). Pre-treated Populus tomentiglandulosa extract inhibits neuronal loss and alleviates gliosis in the gerbil hippocampal CA1 area induced by transient global cerebral ischemia. Anat. Cell Biol..

[8] Song K., Xu L., Zhang W., Cai Y., Jang B., Oh J., Jin J.O. (2017). Laminarin promotes anti-cancer immunity by the maturation of dendritic cells. Oncotarget.

[9] Yang L., Wang L., Zhu C., Wu J., Yuan Y., Yu L., Xu Y., Xu J., Wang T., Liao Z. (2017). Laminarin counteracts diet-induced obesity associated with glucagon-like peptide-1 secretion. Oncotarget.

[10] Shang H.S., Shih Y.L., Chen C.P., Lee M.H., Lu H.F., Chou P.Y., Liao N.C., Chen Y.L., Hsueh S.C., Chung J.G. (2018). Laminarin Promotes Immune Responses and Normalizes Glutamic Oxaloacetic Transaminase and Glutamic Pyruvic Transaminase Levels in Leukemic Mice In Vivo. In Vivo.

[11] Liu X., Liu H., Zhai Y., Li Y., Zhu X., Zhang W. (2017). Laminarin protects against hydrogen peroxide-induced oxidative damage in MRC-5 cells possibly via regulating NRF2. PeerJ.

[12] Yao X., Jiang H., Liang S., Shen X., Gao Q., Xu Y.N., Kim N.H. (2018). Laminarin enhances the quality of aged pig oocytes by reducing oxidative stress. J. Reprod. Dev..

[13] Kim I.H., Lee T.K., Cho J.H., Lee J.C., Park J.H., Ahn J.H., Shin B.N., Chen B.H., Tae H.J., Kim Y.H. (2017). Pretreatment with Chrysanthemum indicum Linne extract protects pyramidal neurons from transient cerebral ischemia via increasing antioxidants in the gerbil hippocampal CA1 region. Mol. Med. Rep..

[14] Lee J.C., Chen B.H., Cho J.H., Kim I.H., Ahn J.H., Park J.H., Tae H.J., Cho G.S., Yan B.C., Kim D.W. (2015). Changes in the expression of DNA-binding/differentiation protein inhibitors in neurons and glial cells of the gerbil hippocampus following transient global cerebral ischemia. Mol. Med. Rep..

[15] Park J.H., Kim I.H., Ahn J.H., Noh Y.H., Kim S.S., Lee T.K., Lee J.C., Shin B.N., Sim T.H., Lee H.S. (2019). Pretreated Oenanthe Javanica extract increases anti-inflammatory cytokines, attenuates gliosis, and protects hippocampal neurons following transient global cerebral ischemia in gerbils. Neural Regen. Res..

[16] Park S.Y., Kim Y.J., Park G., Kim H.H. (2019). Neuroprotective effect of Dictyopteris divaricata extract-capped gold nanoparticles against oxygen and glucose deprivation/reoxygenation. Colloids Surf. B Biointerfaces.

[17] Kim I.H., Yoo K.Y., Park J.H., Yan B.C., Ahn J.H., Lee J.C., Kwon H.M., Kim J.D., Kim Y.M., You S.G. (2014). Comparison of neuroprotective effects of extract and fractions from Agarum clathratum against experimentally induced transient cerebral ischemic damage. Pharm. Biol..

[18] Kang G.H., Yan B.C., Cho G.S., Kim W.K., Lee C.H., Cho J.H., Kim M., Kang I.J., Won M.H., Lee J.C. (2012). Neuroprotective effect of fucoidin on lipopolysaccharide accelerated cerebral ischemic injury through inhibition of cytokine expression and neutrophil infiltration. J. Neurol. Sci..

[19] Kim H., Ahn J.H., Song M., Kim D.W., Lee T.K., Lee J.C., Kim Y.M., Kim J.D., Cho J.H., Hwang I.K. (2019). Pretreated fucoidan confers neuroprotection against transient global cerebral ischemic injury in the gerbil hippocampal CA1 area via reducing of glial cell activation and oxidative stress. Biomed. Pharm..

[20] Alvarez J.I., Katayama T., Prat A. (2013). Glial influence on the blood brain barrier. Glia.

[21] Cabezas R., Avila M., Gonzalez J., El-Bacha R.S., Baez E., Garcia-Segura L.M., Jurado Coronel J.C., Capani F., Cardona-Gomez G.P., Barreto G.E. (2014). Astrocytic modulation of blood brain barrier: Perspectives on Parkinson’s disease. Front. Cell. Neurosci..

[22] Michinaga S., Koyama Y. (2019). Dual Roles of Astrocyte-Derived Factors in Regulation of Blood-Brain Barrier Function after Brain Damage. Int. J. Mol. Sci..

[23] Ohmori C., Sakai Y., Matano Y., Suzuki Y., Umemura K., Nagai N. (2018). Increase in blood-brain barrier permeability does not directly induce neuronal death but may accelerate ischemic neuronal damage. Exp. Anim..

[24] Dheen S.T., Kaur C., Ling E.A. (2007). Microglial activation and its implications in the brain diseases. Curr. Med. Chem..

[25] Jayaraj R.L., Azimullah S., Beiram R., Jalal F.Y., Rosenberg G.A. (2019). Neuroinflammation: Friend and foe for ischemic stroke. J. Neuroinflamm..

[26] Hwang I.K., Park J.H., Lee T.K., Kim D.W., Yoo K.Y., Ahn J.H., Kim Y.H., Cho J.H., Kim Y.M., Won M.H. (2017). CD74-immunoreactive activated M1 microglia are shown late in the gerbil hippocampal CA1 region following transient cerebral ischemia. Mol. Med. Rep..

[27] Daneman R., Prat A. (2015). The blood-brain barrier. Cold Spring Harb. Perspect. Biol..

[28] Banerjee S., Bhat M.A. (2007). Neuron-glial interactions in blood-brain barrier formation. Ann. Rev. Neurosci..

[29] Muresanu D.F., Sharma A., Patnaik R., Menon P.K., Mossler H., Sharma H.S. (2019). Exacerbation of blood-brain barrier breakdown, edema formation, nitric oxide synthase upregulation and brain pathology after heat stroke in diabetic and hypertensive rats. Potential neuroprotection with cerebrolysin treatment. Int. Rev. Neurobiol..

[30] Stranahan A.M., Hao S., Dey A., Yu X., Baban B. (2016). Blood-brain barrier breakdown promotes macrophage infiltration and cognitive impairment in leptin receptor-deficient mice. J. Cereb. Blood Flow Metab..

[31] Ahn J.H., Chen B.H., Park J.H., Shin B.N., Lee T.K., Cho J.H., Lee J.C., Park J.R., Yang S.R., Ryoo S. (2018). Early IV-injected human dermis-derived mesenchymal stem cells after transient global cerebral ischemia do not pass through damaged blood-brain barrier. J. Tissue Eng. Regen. Med..

[32] Zhang H., Park J.H., Maharjan S., Park J.A., Choi K.S., Park H., Jeong Y., Ahn J.H., Kim I.H., Lee J.C. (2017). Sac-1004, a vascular leakage blocker, reduces cerebral ischemia-reperfusion injury by suppressing blood-brain barrier disruption and inflammation. J. Neuroinflamm..

[33] Ha Park J., Yoo K.Y., Hye Kim I., Cho J.H., Lee J.C., Hyeon Ahn J., Jin Tae H., Chun Yan B., Won Kim D., Kyu Park O. (2016). Hydroquinone Strongly Alleviates Focal Ischemic Brain Injury via Blockage of Blood-Brain Barrier Disruption in Rats. Toxicol. Sci..

[34] Chang R.C., So K.F. (2008). Use of anti-aging herbal medicine, Lycium barbarum, against aging-associated diseases. What do we know so far?. Cell. Mol. Neurobiol..

[35] Yang D., Li S.Y., Yeung C.M., Chang R.C., So K.F., Wong D., Lo A.C. (2012). Lycium barbarum extracts protect the brain from blood-brain barrier disruption and cerebral edema in experimental stroke. PLoS ONE.

[36] Carpenter T.S., Kirshner D.A., Lau E.Y., Wong S.E., Nilmeier J.P., Lightstone F.C. (2014). A method to predict blood-brain barrier permeability of drug-like compounds using molecular dynamics simulations. Biophys. J..

[37] Mitchell D.M., Sun C., Hunter S.S., New D.D., Stenkamp D.L. (2019). Regeneration associated transcriptional signature of retinal microglia and macrophages. Sci. Rep..

[38] Colonna M., Butovsky O. (2017). Microglia Function in the Central Nervous System During Health and Neurodegeneration. Ann. Rev. Immunol..

[39] Suzumura A. (2013). Neuron-microglia interaction in neuroinflammation. Curr. Protein Pept. Sci..

[40] Gulke E., Gelderblom M., Magnus T. (2018). Danger signals in stroke and their role on microglia activation after ischemia. Ther. Adv. Neurol. Disord..

[41] Yoo K.Y., Yoo D.Y., Hwang I.K., Park J.H., Lee C.H., Choi J.H., Kwon S.H., Her S., Lee Y.L., Won M.H. (2011). Time-course alterations of Toll-like receptor 4 and NF-kappaB p65, and their co-expression in the gerbil hippocampal CA1 region after transient cerebral ischemia. Neurochem. Res..

[42] Schilling M., Besselmann M., Muller M., Strecker J.K., Ringelstein E.B., Kiefer R. (2005). Predominant phagocytic activity of resident microglia over hematogenous macrophages following transient focal cerebral ischemia: An investigation using green fluorescent protein transgenic bone marrow chimeric mice. Exp. Neurol..

[43] Cai Q., Li Y., Pei G. (2017). Polysaccharides from Ganoderma lucidum attenuate microglia-mediated neuroinflammation and modulate microglial phagocytosis and behavioural response. J. Neuroinflamm..

[44] Teng P., Li Y., Cheng W., Zhou L., Shen Y., Wang Y. (2013). Neuroprotective effects of Lycium barbarum polysaccharides in lipopolysaccharide-induced BV2 microglial cells. Mol. Med. Rep..

[45] Lee T.K., Park J.H., Ahn J.H., Kim H., Song M., Lee J.C., Kim J.D., Jeon Y.H., Choi J.H., Lee C.H. (2019). Pretreatment of Populus tomentiglandulosa protects hippocampal CA1 pyramidal neurons from ischemia-reperfusion injury in gerbils via increasing SODs expressions and maintaining BDNF and IGF-I expressions. Chin. J. Nat. Med..

[46] Carpenter J.W., Marion C.J. (2013). Exotic animal formulary.

[47] Park J.H., Ahn J.H., Song M., Kim H., Park C.W., Park Y.E., Lee T.K., Lee J.C., Kim D.W., Lee C.H. (2019). A 2-Min Transient Ischemia Confers Cerebral Ischemic Tolerance in Non-Obese Gerbils, but Results in Neuronal Death in Obese Gerbils by Increasing Abnormal mTOR Activation-Mediated Oxidative Stress and Neuroinflammation. Cells.

[48] Kim H., Park J.H., Shin M.C., Cho J.H., Lee T.K., Song M., Park C.W., Park Y.E., Lee J.C., Ryoo S. (2019). Fate of Astrocytes in The Gerbil Hippocampus After Transient Global Cerebral Ischemia. Int. J. Mol. Sci..

[49] Park J.H., Ahn J.H., Kim D.W., Lee T.K., Park C.W., Park Y.E., Lee J.C., Lee H.A., Yang G.E., Won M.H. (2019). Altered Nurr1 protein expression in the hippocampal CA1 region following transient global cerebral ischemia. Mol. Med. Rep..

